# Extramedullary Leukemia, Presenting at the Cervix of the Uterus

**DOI:** 10.1155/2020/8492036

**Published:** 2020-08-31

**Authors:** Nikolaos Tsagkas, Androniki Troussa, George Vorgias, Olympia Tzaida, Nektaria Zagorianakou, Maria Gonidi

**Affiliations:** ^1^Obstetrics and Gynecology, General Hospital of Lefkas, Aristoteli Valaoriti 29, 31100 Lefkas, Greece; ^2^Private Practice, Obstetrics and Gynecology, Corfu, Greece; ^3^Gynecology Clinic of Metaxa Cancer Hospital of Piraeus, Botasi 51, 18537 Athens, Greece; ^4^Pathology Department of Metaxa Cancer Hospital of Piraeus, Botasi 51, 18537 Athens, Greece; ^5^Private Practice, Cytopathology Laboratory, Ioannina, Greece; ^6^Private Practice, Cytopathology Laboratory, Corfu, Greece

## Abstract

Extramedullary relapse of leukemia is encountered more often than in the past. The reason is that leukemia survival rates increase with improved treatment schemes. We present a rare case of involvement of the cervix of the uterus in an adult B Acute Lymphocytic Leukemia (B-ALL) survivor. Relapses affect various organs but rarely the female genital tract. Nevertheless, in this case, a woman with a history of induced amenorrhea due to treatment for leukemia presented to the gynecologist because of vaginal spotting. Colposcopy evaluation of the vagina/cervix, sonography and cytological and histological sampling established the diagnosis of leukemia relapse in the cervix of the uterus. Under these circumstances, our study highlights the rare extramedullary presentation of leukemia in the cervix of the uterus of a young lady considered to be disease-free and listed for bone marrow transplantation. In this rare case of relapse in the cervix of the uterus, Pap smears alarmed physicians, and radiology examinations assisted the diagnostic workup. Still, only biopsy, microscopic evaluation, and immunohistochemistry studies established the exact diagnosis. Prognosis in the situation of extramedullary disease relapse in the female genital tract was poor, but gynecologists' high suspicion led to a prompt diagnosis. Survival is in general limited, but together with high suspicion, multidisciplinary team involvement is imperative to improve the reduced chances of survival.

## 1. Case Presentation

A young nulliparous 30-year-old female presented with vaginal (mid cycle) spotting. Her last gynecological assessment had been unremarkable, with the most recent cervical smear—5 years ago—being negative for intraepithelial lesion or malignancy. Interestingly, the patient was on the waiting list for graft transplantation and an appropriately matched donor had been already identified.

During the last five years and following front-line chemotherapy, the patient relapsed twice. Nevertheless, over the previous 12 months, the young lady was considered to be in complete remission (CR). By CR it means that there is no evidence of leukemia, which implies that the following criteria are met: (a) normal physical findings, (b) peripheral blood microscopy—normal, (c) <5% of blasts in the BM, and (d) absence of detectable central nervous system (CNS) or other extramedullary (EM) disease. In the case of relapsed ALL patients in CR, the hematooncologic best practice is considered to be Hematopoietic Stem Cell Transplantation (HSCT) and this therapeutic plan was followed in the current case. The scheme of pretransplantation chemotherapy and total body irradiation was initiated in preparation for hosting the bone marrow graft. Due to the treatment, secondary amenorrhea has been induced; however, the patient visited her gynecologist due to vaginal spotting.

The standard gynecological examination, as a means of annual preventive strategy, typically includes a visual inspection of the external genitalia, vagina, and the cervix through a speculum. Moreover, transvaginal scans (TVS) were also performed.

**Figure 1:**
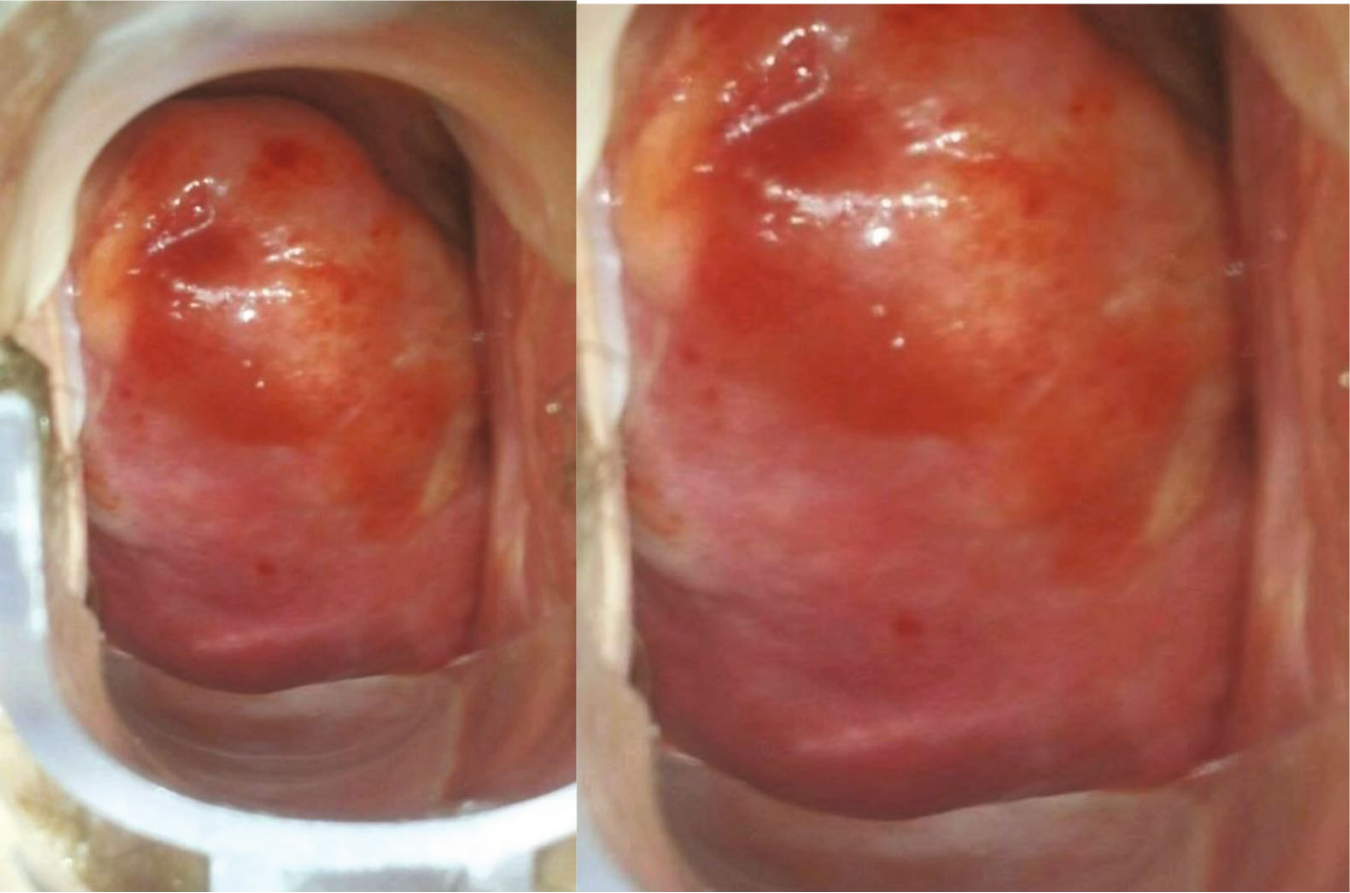
Colposcopy.

**Figure 2:**
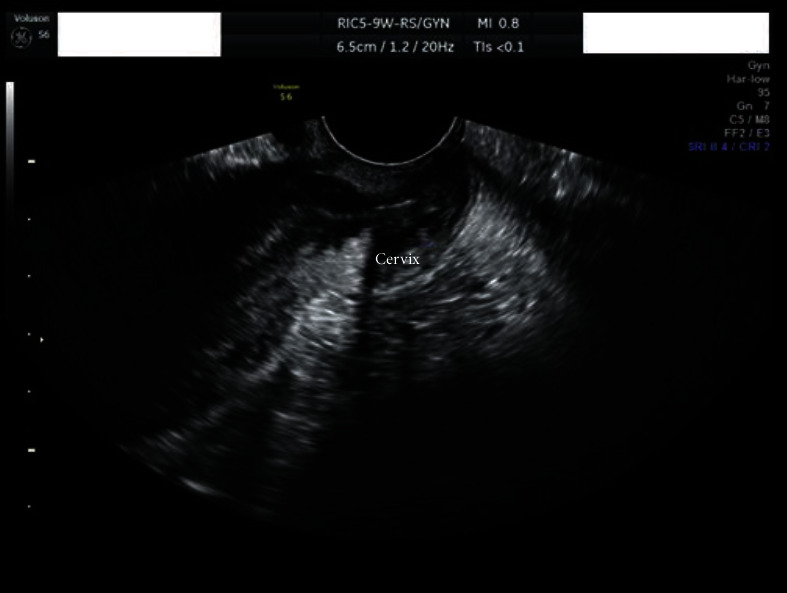
Transvaginal scan image.

**Figure 3:**
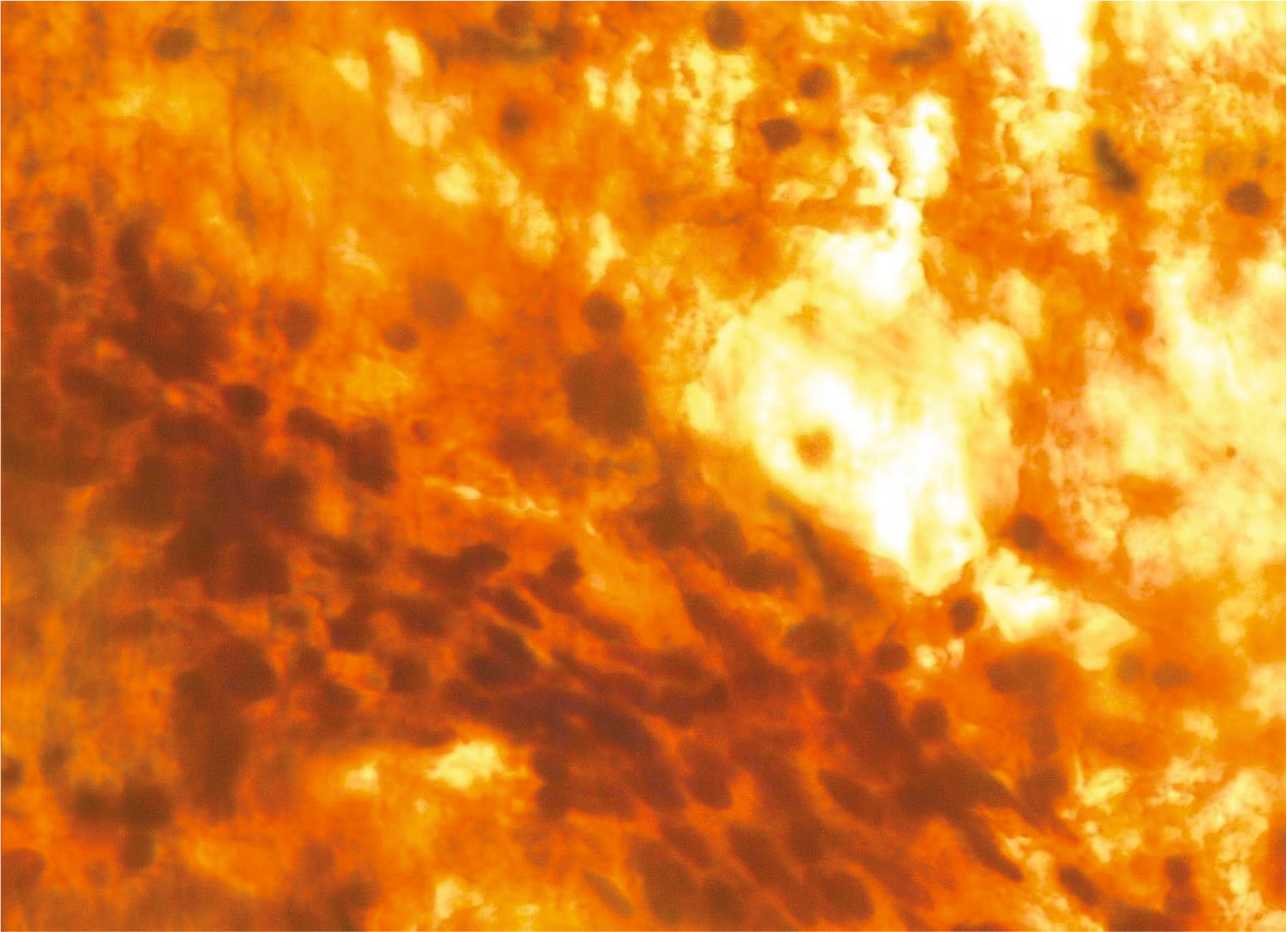
Pap smear.

**Figure 4:**
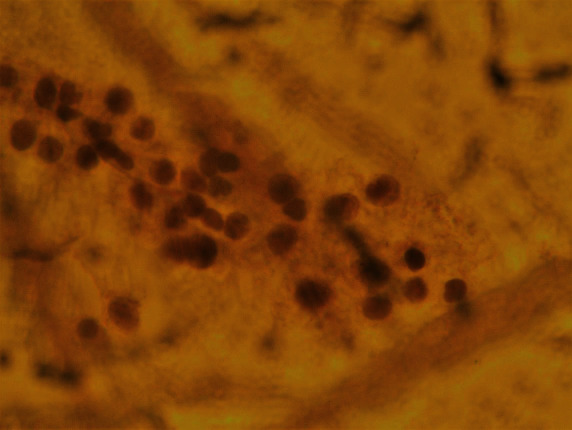
Pap smear.

**Figure 5:**
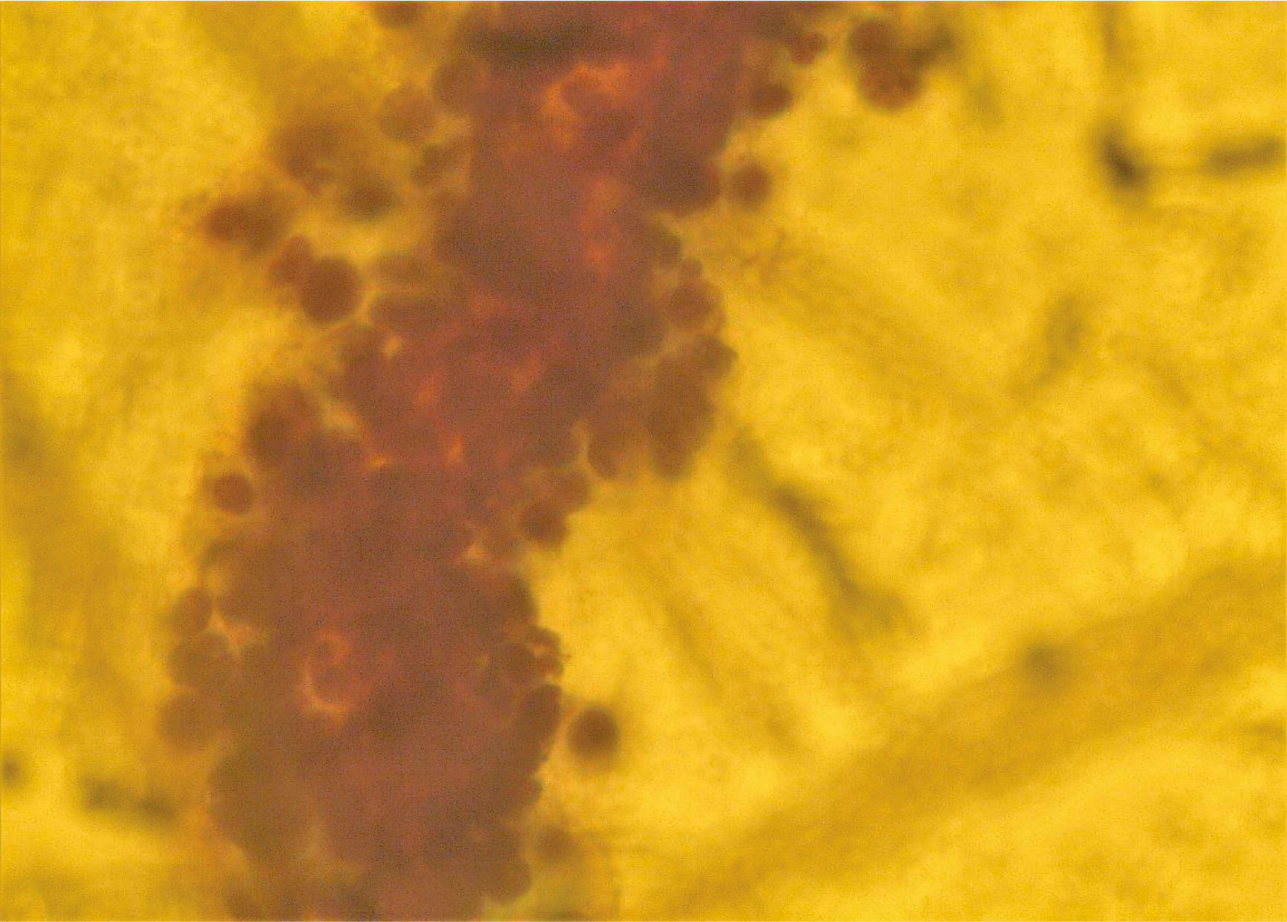
Pap smear.

**Figure 6:**
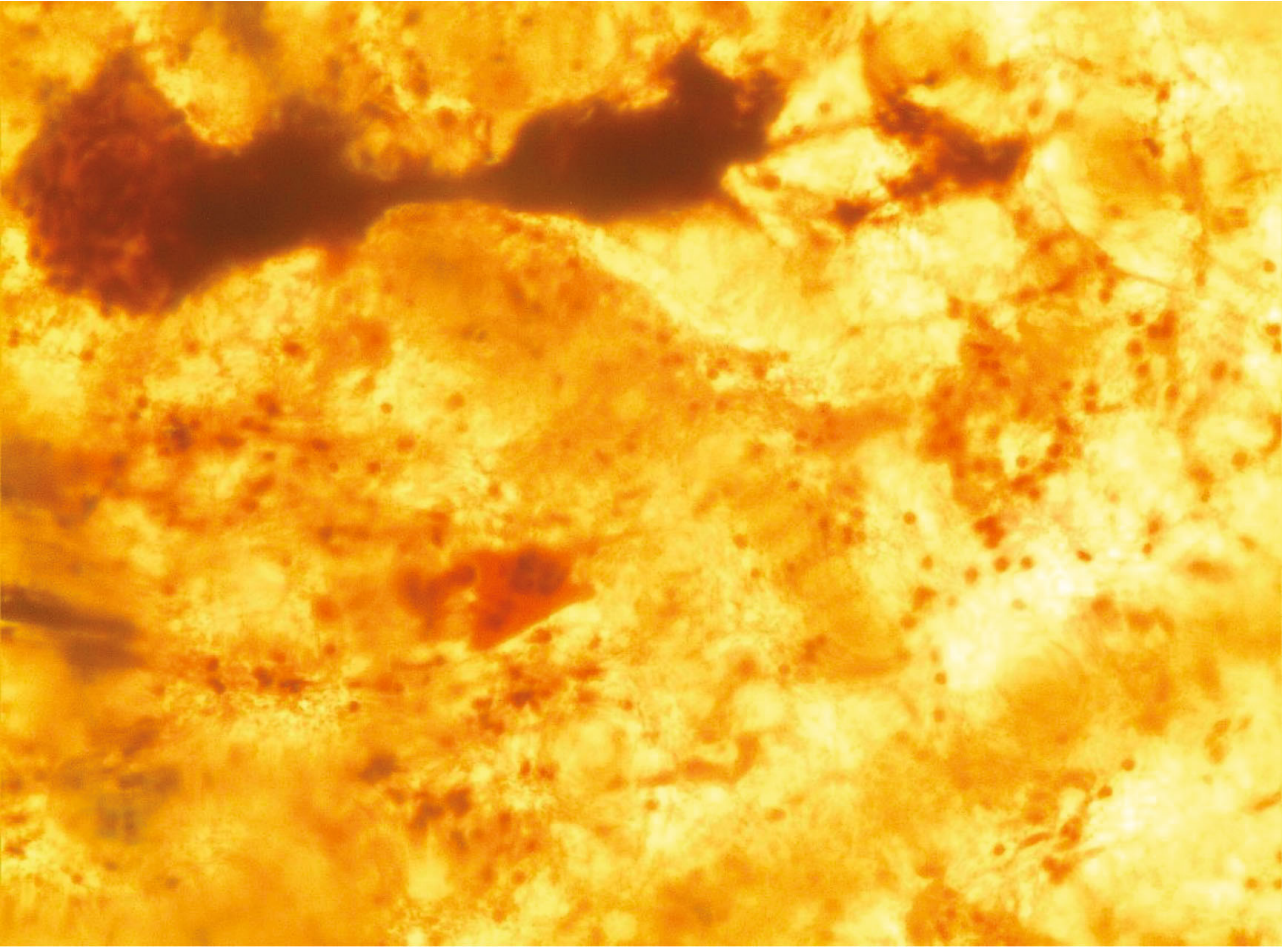
Pap smear.

**Figure 7:**
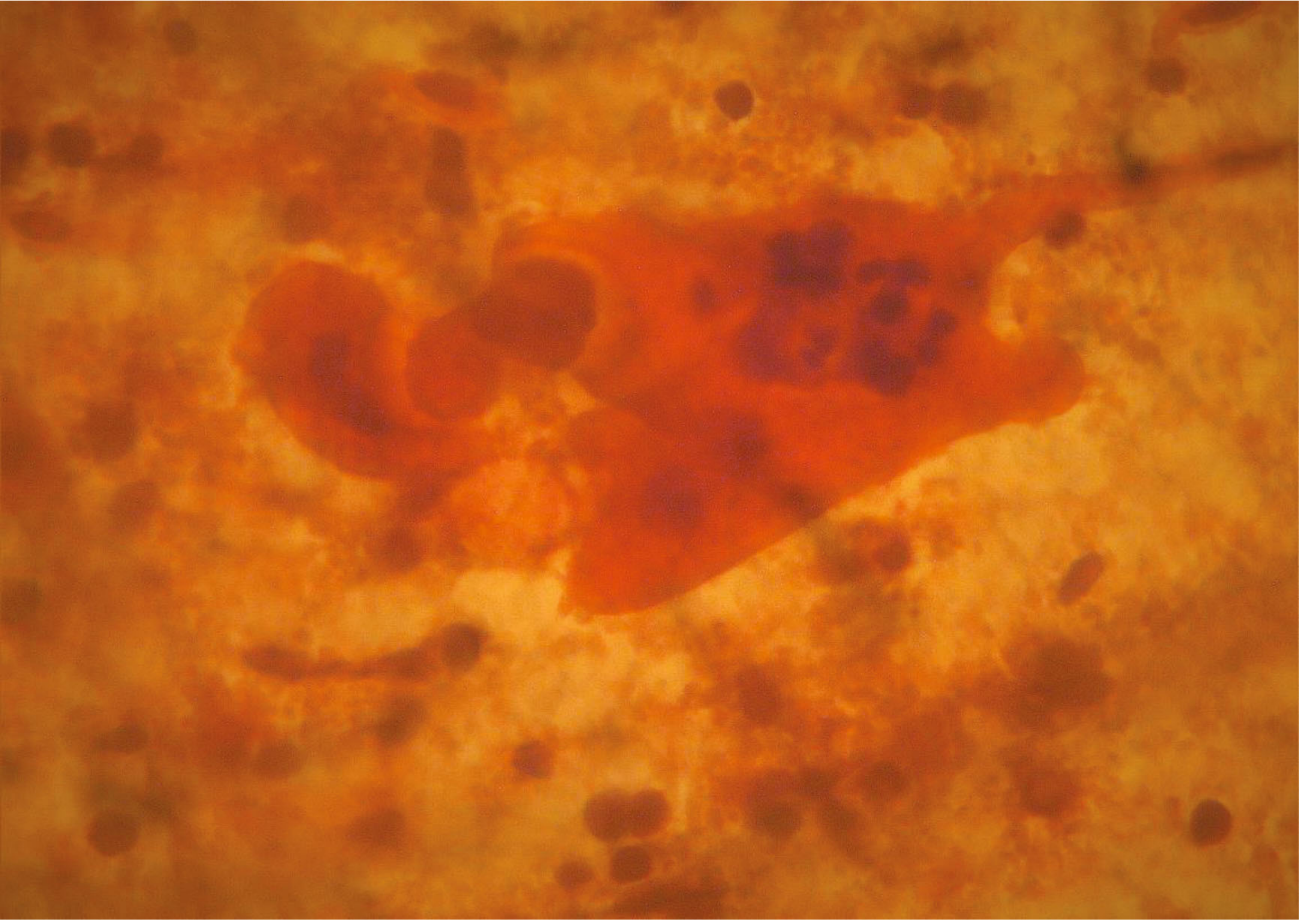
Pap smear.

On speculum examination, the outer two-thirds of the vagina and the cervix at the site of the transformation zone appeared normal. However, at the posterior fornix of the vagina, a “discolored area” was seen, extending to the cervix but not reaching the transformation zone and the cervical orifice. There were no obvious signs of laceration, bleeding, or neoplastic erosion of the epithelium. However, it drew the clinical suspicion of the attending gynecologist as an “atypical lesion” which necessitated further thorough evaluation (Figure 1). The impression at first was of an “enlarged/stony cervix at the bimanual examination,” mimicking a common benign condition such as a cervical fibroid. For completion purposes, a TVS was performed. Analogously with the speculum inspection, TVS revealed at the site of the “discolored lesion” a hyperechogenic area and the relevant acoustic shadow was depicted (Figure 2). At the same visit, samples were taken for cytological examination, including smears from the cervical OS, aimed at evaluating the transformation zone, and smears from the site of the lesion. According to the cytological report, the cytological sample reflected “infiltration of the cervix by lymphoblastic cells” (Figures 3–7).

**Figure 8:**
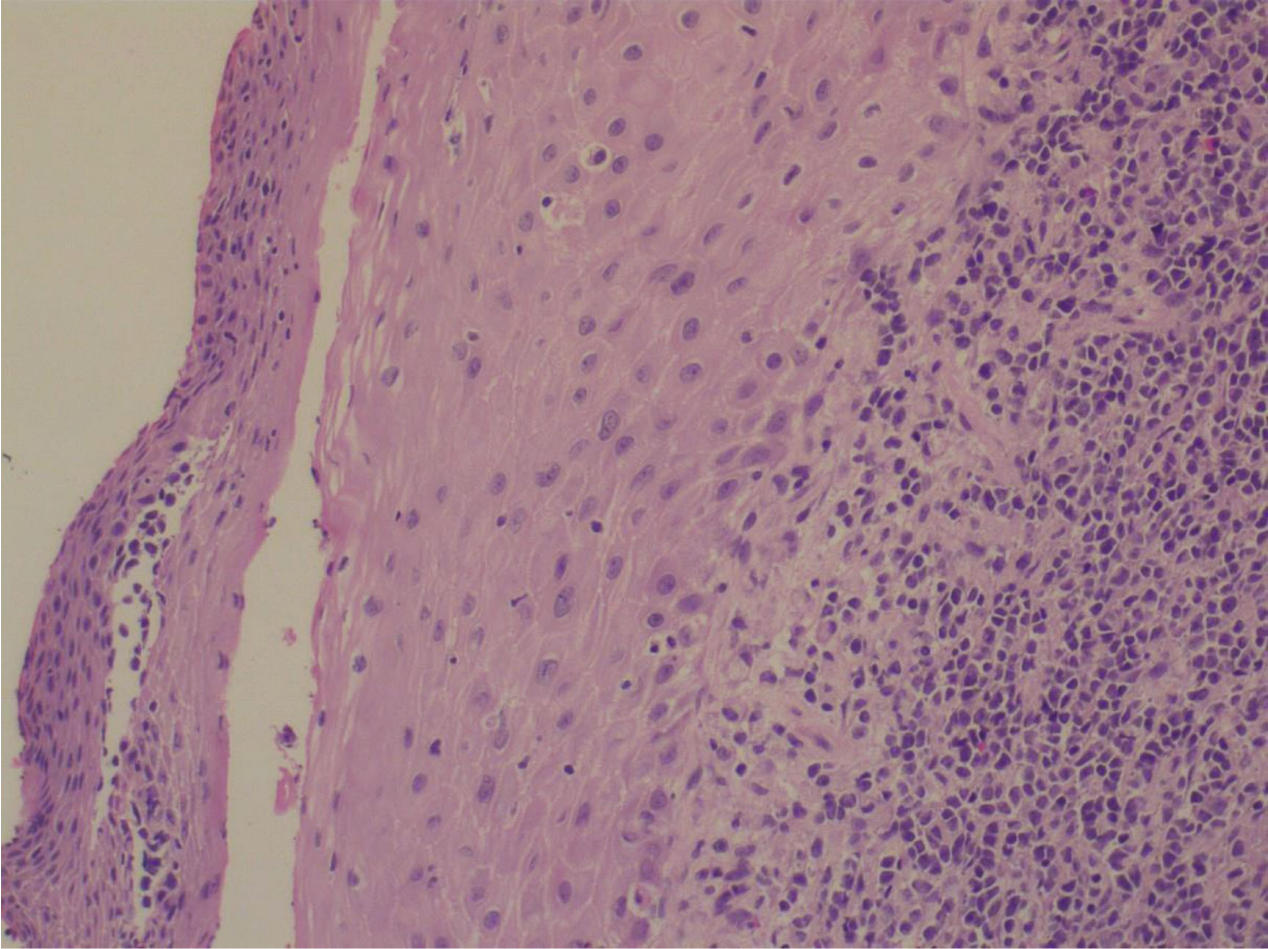
Histology.

**Figure 9:**
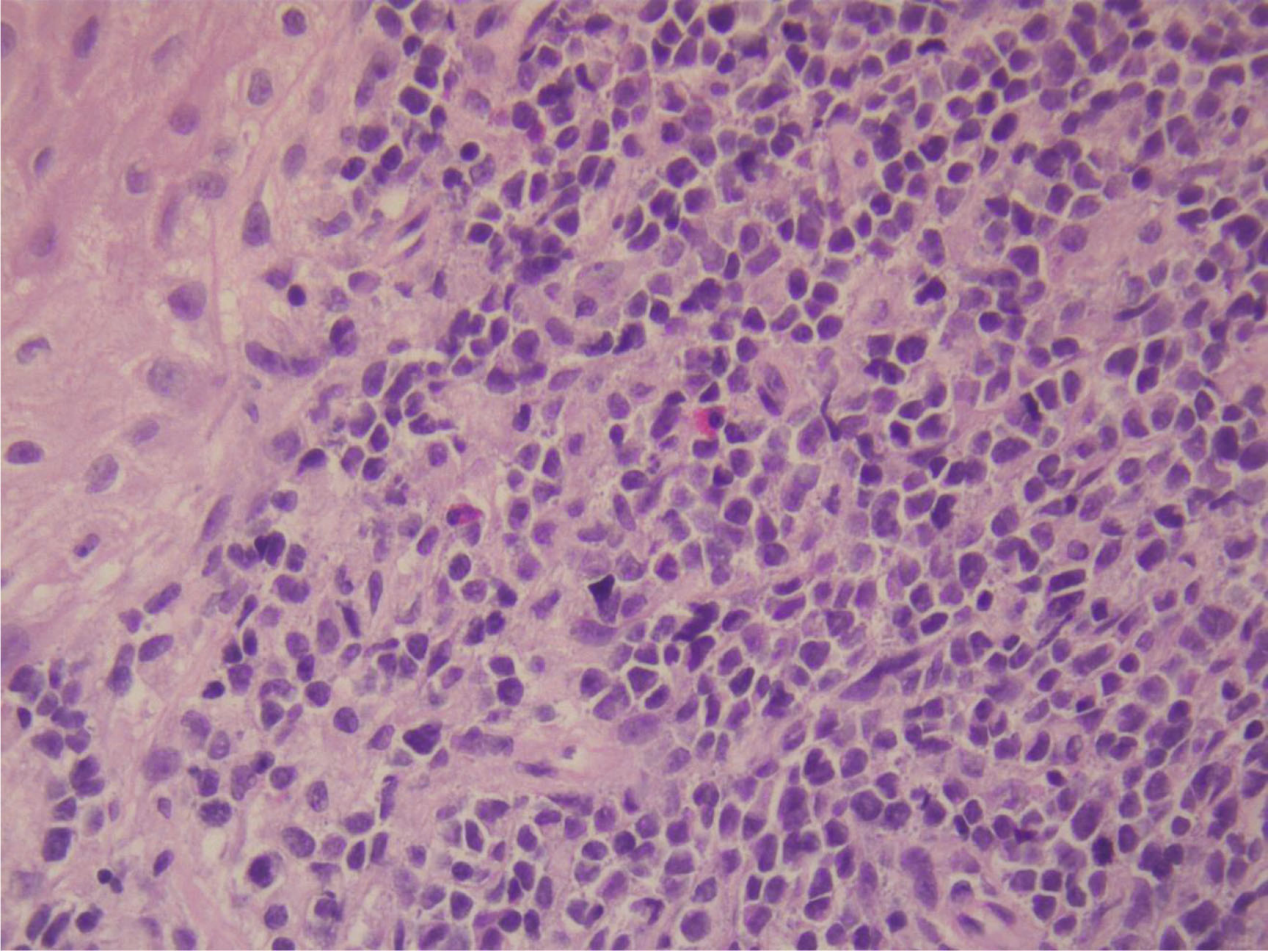
Histology.

The patient had further consultation to discuss the details of the management plan. Punch biopsies for histological confirmation and further evaluation were decided. The histological result confirmed both the clinical impression and the cytological result leading to the diagnosis of “infiltration of the cervix by leukemic blasts” (Figures 8 and 9).

**Figure 10:**
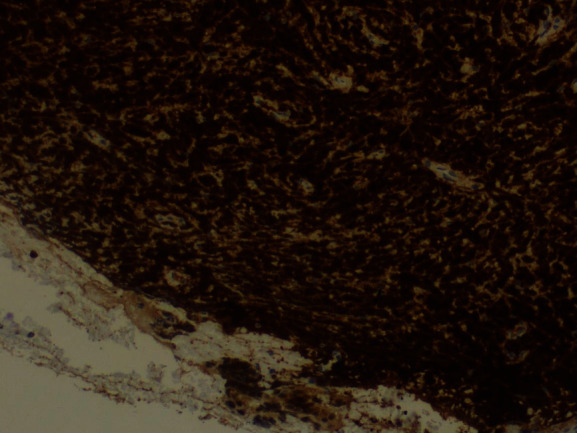
*MIB* 1/Ki-67: positive 100%.

**Figure 11:**
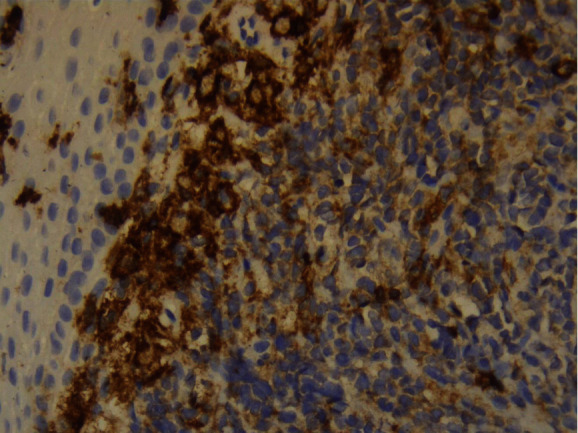
CD34: weak positive.

**Figure 12:**
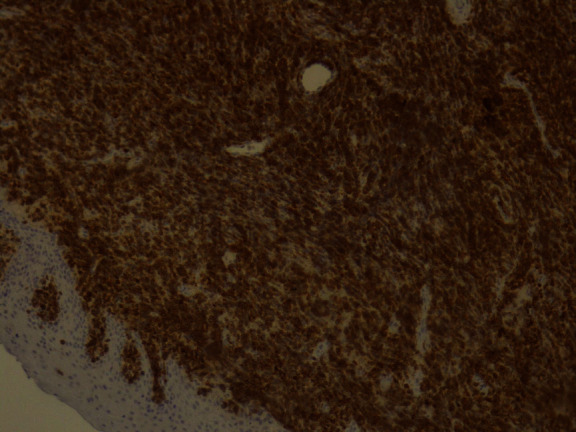
CD45: positive.

**Figure 13:**
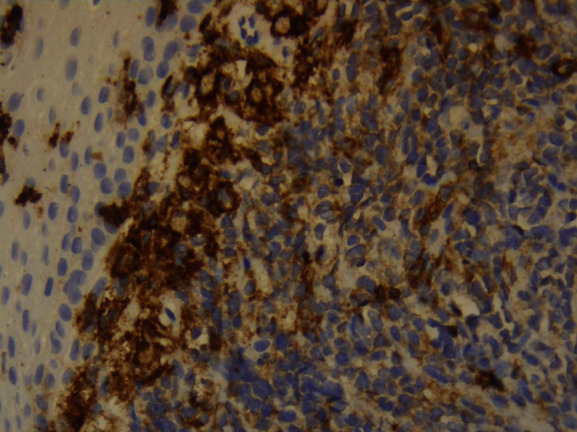
CD20: weak positive.

**Figure 14:**
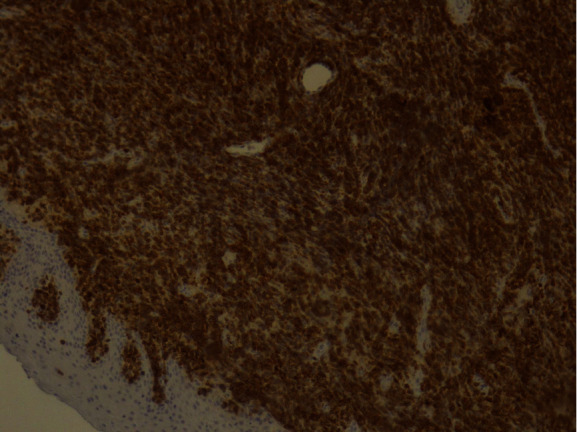
CD79A: positive.

**Figure 15:**
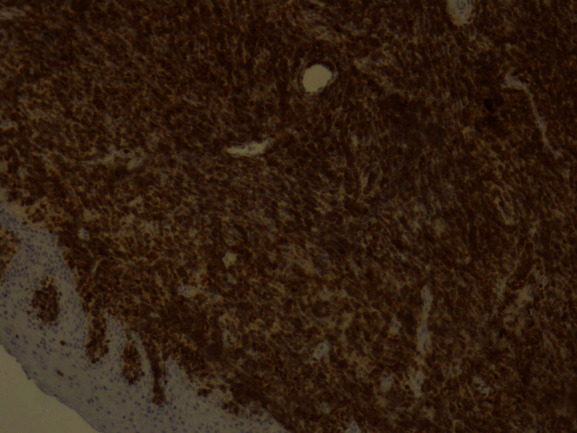
CD10: positive.

The tumor surface molecules were studied by immunophenotyping (Table 1) to clarify the nature of cancer and establish the appropriate treatment protocol. The positive Clusters of Differentiation (CD) were CDs 34, 45, 20, 79A, and 10 together with the marker MIB 1/Ki-67 (Figures 10–15).

Following that, the diagnosis of disease relapse was established with extramedullary localization of leukemia. Specifically, the case presented refers to the rare finding of “nonprimary cervical disease—lymphoblastic leukemia, presenting with cervical pathology.”

## 2. Summary

A nulliparous lady in her early thirties, with a history of induced amenorrhea due to treatment for leukemia, presented to the gynecologist because of vaginal spotting.

It was the gynecologist's clinical suspicion and the thorough gynecological examination, together with the use of diagnostic adjuncts such as colposcopy visualization of the vagina/cervix and sonography, which led to further characterization. A diagnostic pathway was planned, including cytological and histological sampling, and the relevant results established the diagnosis of leukemia relapse with the rare presentation of a lesion at the cervix of the uterus.

A holistic approach is essential when assessing each patient and specifically those with chronic disease. A multidisciplinary team strategy is of paramount importance, as rare presentations of severe systematic disorders can be diagnosed promptly.

The current case highlights the rare extramedullary presentation of leukemia in the cervix of the uterus of a young lady considered to be disease-free and listed for bone marrow transplantation.

## 3. Discussion

### 3.1. Sites of Extramedullary Relapse of ALL

The majority of adult patients with ALL (60-90%) will reach complete remission after initial therapy, but relapses occur often. 1/3 of ALL patients with standard risk ALL and 2/3 of high-risk ALL patients will experience a relapse. High-risk ALL patients are those characterized with the following: WBC > 30 × 10^9^/L, >30 years old, and unfavorable cytogenetic features (*t*(9; 22), *t*(1; 19), or *t*(4; 11)) [1, 2].

Patients who relapse are more challenging to treat. Regardless of the type of initial treatment (bone marrow transplantation or chemotherapy), the one-year survival is 22%, and the five-year survival is only 7% according to well-designed studies. The only chance for increased survival for relapsed adult ALL patients is via allogeneic HSCT after reaching a second complete remission phase. Furthermore, it is highly recommended for the patients to be offered a clinical trial (e.g., studying monoclonal antibodies or novel chemotherapeutic agents) since current treatments are not effective enough to be considered as salvage treatments [3].

Most of the cases of relapse include infiltration of the medulla. Nevertheless, in a few rare cases, the relapses are solely located in extramedullary organs (extramedullary relapse (EMR)), with only ~8% of relapses being EMR alone. As for the relapse time frame, the data from UKALL XII/ECOG show that they frequently occur within two years (81%) and the remaining 19% relapse two years postdiagnosis [3].

The extramedullary organs which present as lymphoblastic cell sanctuaries at disease reappearance are those of the central nervous system (CNS) in both sexes, and the testes are the second site of disease reappearance in men [1].

In literature review, cases of rare appearances of EMR can be found. Those include several sites such as infiltration of the liver, pancreas, kidneys, breasts, bones, and soft tissue/muscles and gastric organ infiltration with lymphoblasts [4–8].

### 3.2. Involvement of the Gynecologic Organs

As a general rule for leukemia, EMRs are rather expected in cases of Acute Myeloblastic Leukemia (AML) than in ALL patients [5].

Particularly for the female genital tract, lymphoblastic infiltration is rare in hematooncological diseases, and gynecologic pelvic pathology is only expected in lymphomas. Nevertheless, it still comprises a rare minority as <1% of primary extranodal lymphomas are located in female genital organs, affecting in this case mainly the cervix and less frequently the ovaries and the uterine corpus [6, 7].

#### 3.2.1. Ovary Infiltration in ALL

Regarding adnexal involvement, Narang et al. (2016) described a case of a 20-year-old woman with ALL and primary involvement of the ovaries. The young patient was referred to the hematologist after undergoing abdominal hysterectomy and bilateral salpingo-oophorectomy. The surgery was performed for treating what was thought to be a tuboovarian mass, formed by a complicated ruptured ectopic and impending sepsis (vaginal bleeding and thrombocytopenia, PLT = 32.000) [8].

The second peak of ALL is at the age of 50 years, and Sahu et al. (2016) analyzed the case of a 47-year-old lady with known ALL (precursor B cell) who presented with complaints of “vague sensation” in the lower abdomen. Blood tests were within normal limits, and physical examination did not reveal any other pathological finding except a palpable mass at the right iliac fossa. The computed tomography depicted a mass 8 × 6 × 8 cm at the right adnexa, and the differentials included ovarian cancer or ALL relapse. Fine-needle aspiration from the lesion and biopsies from the uterine cervix and the bone marrow revealed systematic ALL relapse with concurrent infiltration of the gynecological organs. Complete resolution followed after 30 days in chemotherapy treatment, and HSCT was planned [9].

Gonadal infiltration is often a necropsy finding and rarely a clinical entity. Accordingly and to detect EMRs early, the authors suggest that at least biannual gynecologic pelvic examination should be included in regular follow-up of adult females after completion of therapy for ALL aimed at detecting EMRs in a limited early stage. It has to be noted that routinely after completion of the maintenance phase of therapy, ALL patients are followed every 3-4 months with a detailed physical exam and haemogram [10, 11].

The fact that postmortem autopsies show a higher than thought incidence of lymphocytic infiltration in women with known leukemia allows correlation of the ovaries with their male equivalent the testes. The testes are well-known sites of lymphocytic infiltration. Firstly the CNS and secondly the testes are considered to be lymphocytic sanctuaries, in other words, as anatomic sites which are poorly penetrated by chemotherapeutic standard agents for ALL, thus posing an important threat for ALL relapse ignition [12]. It is known that the ovaries share a common embryological ancestor with the testes, and postmortem studies show that the incidence of ovarian leukemic infiltrates in necropsy reports of patients with leukemia rises to 36% (29–92% incidence for the testes) [13, 14]. This may implicate that the ovaries similarly to the testes represent an undervalued sanctuary site for leukemia in females.

#### 3.2.2. Vagina Infiltration in ALL

The adnexa are not the only FGT spot at which infiltrates develop.

Another interesting report by Sava et al. (2019) refers to the case of a 15-year-old young lady who presented with a vaginal mass (EMR, lymphoblast infiltration) 17 months after the end of the maintenance therapy for ALL. The patient was treated with chemotherapy, the mass regressed in 9 months, and HSCT was planned. Before HSCT, a retroperitoneal EMR was also treated with chemotherapeutic regimes, and post HSCT, the third EMR came up developing as lymphoblastic infiltration of the gastric wall. Death occurred due to disease progression after ten years from the initial diagnosis [15].

Relapse developing as a vaginal mass was reported by Qamruddin et al. (1995) as well, in an even younger girl (eight years old). Two years postremission while in maintenance therapy, the young patient complained of urinary retention. A friable mass easily bleeding when touched was biopsied at the upper vagina, and this finding represented a leukemic cell infiltrated lump [13].

#### 3.2.3. Uterine Corpus Infiltration in ALL

Except for the presentation of palpable masses, there are also signs such as vaginal spotting. Vaginal spotting is a nonspecific sign which frequently manifests female genital tract pathology. Specifically, uterine bleeding caused by leukemia is an entity that is explained by the following mechanisms: (1) thrombocytopenia—impaired hemopoiesis and (2) functional leukemic endometrial bleeding. By the previous term, the meaning is that the dense leukemic infiltration leads to extensive parenchymal destruction of the endometrium, thus predisposing in AUB.

Robillard et al. (2017) reported the case of a 12-year-old girl with vaginal spotting of the uterine corpus. The girl was treated with chemoradiation and allogeneic bone marrow transplantation. Following the initial treatment, she kept on bleeding through the vagina every day for two months. Ultrasound revealed an enlarged uterus (the size of 14 weeks of pregnancy), and biopsies established the diagnosis of EMR of beta lymphocytic leukemia. The uterus regressed to normal size by resuming medical treatment for leukemia [16].

The first report of leukemic infiltration of the endometrium by precursor B cell ALL is documented in 1939 by McDonald and Waugh [17]. In a 1998 report, a 5-year-old CD10 positive, in remission for 2 and 1/2 years, relapsed with uterine mass. Reinduction therapy and local radiation were initiated [18].

Other than solely infiltrating the uterine corpus, in literature, cases of combined leukemic infiltration exist. Zutter and Gersell in 1990 reported a relapse of T-ALL, localized at the uterus and cervix after two years of disease-free interval (CD1, 2, 5, 4, 7, and 8 positive) [19]. Similarly, Lyman and Neuhauser in 2002 referred to a 38-year-old T-ALL patient, being in remission for four years, who relapsed with uterine, cervical, and appendicular leukemic infiltration. Her survival was limited to 10 months after relapse was diagnosed [20].

#### 3.2.4. Cervix of the Uterus Infiltration in ALL

The case presented in the current report refers to the rare case of ALL relapse in the gynecologic organs and specifically in the cervix of the uterus. Similarly, other authors have analyzed analogous cases, for example, a 42-year-old woman with a primary presentation of ALL. In biopsy of a polypoid growth that was protruding from the cervical OS, leukemic cells were found to be infiltrating the endocervix. This was found after she presented to the gynecologists suffering from menorrhagia for two months. Her treatment included chemotherapy according to the BFM 90 protocol, and complete remission was achieved for >10 years = very long survivor [17].

In another case of ALL in childhood, relapse was diagnosed by cervical smear cytology and immunohistochemistry (IHC) 43 months after the initial diagnosis of ALL. Chemotherapy was the treatment in this case as well, and the girl remained in remission for 54 months [21].

At the other end of the bimodal incidence curve of ALL, a lady at the age of 59 years relapsed with the disease appearing at the cervix of the uterus. Again, the diagnosis was put by cytology from smear taken from the lymphocytic cervical mass (ALL TdT, CD10, 34, 79A positive) [22].

The case report analysis is added to the already existing set of cases of EMR of ALL at the cervix of the womb. The clinical presentation, diagnostic pathway, colposcopy images, and sonographic pictures are resources which can sensitize the gynecologists when assessing patients with a history of hematologic malignancy. In addition, gynecologists should be notified by such cases towards the possibility of an unusual presentation of systematic diseases at the gynecological organs similarly to ALL EMR at the cervix.

### 3.3. Immunohistochemistry

Nowadays, proper diagnosis, classification of disease, and targeted therapeutic plans are based both on the classic morphologic features on microscopic biopsy examination and appropriate molecular adjuncts. Those adjuncts are appropriately selected immunophenotypic panels for tissue profiling and molecular genetics. It is important to mention, though, that there is no pathognomonic marker for a specific disease, and this explains that in each pathology study different immunophenotypic panels are selected [23].

In the case of assessing cervical biopsies, pathologists frequently use the marker Ki-67/MIB 1, as its expression represents “increased cell proliferation.” Furthermore, specifically at cervical pathological assessment, Ki-67 positivity is linked to poor prognosis in cervical cancer [24].

The neoplasm of the case presented exhibited immature characteristics; thus, CD34 and CD45 markers were used. At first, the tissue is stained CD34 positive in the case of hematopoietic disease when undifferentiated blasts are present (85–90% of B-ALL and B lymphoblastic lymphomas). Secondly, for CD45, a strong expression is seen in non-Hodgkin lymphomas; a weak expression is seen in acute leukemias (ALL, AML, and blastic natural killer cell neoplasms). In our case, the expression was positive, and this fact confirms the hematopoietic origin of the cervical tumor [23].

After recognizing the hematopoietic origin, the use of the marker CD20 was warranted because of the history of leukemia and the leukemic cell appearance at the cytology report. CD20 is negative or weakly positive in B-ALL and B lymphoblastic lymphoma and is used as screening for justifying that a tumor derives from B cell lineage since CD20 is negative in T-ALL and AML [23].

Furthermore, positivity for CD20 is considered as a good prognostic factor. This is because therapeutic anti-CD20 agents have been developed and are available as treatment regimes. The medications based on CD20 are rituximab, ibritumomab, and tositumomab which have been used in the successful treatment of both B-ALL and Burkitt leukemia and lymphoma [25].

In addition to the CD20, CD79A is also a B-lineage marker. In the current case, CD79A was strongly expressed as it is expressed in the majority of B-ALL (80% of cases). On the other hand, it is only rarely expressed in T-ALL.

In the same manner, CD10 was strongly expressed, favoring lymphoblastic leukemia. CD10 is not a positive feature of immunohistochemistry in the case of AML; usually, it is expressed in B-ALL and less frequently in T-ALL [23].

### 3.4. Take-Home Messages

The signs and symptoms of extramedullary leukemia in the gynecological organs are nonspecific. The most common complaint is abnormal uterine bleeding/spotting/menorrhagia. The cause could be either thrombocytopenia in patients with acute leukemia or bleeding which could be caused by tissue destruction due to leukemic infiltration [21, 22].

In addition, specialists in the field of gynecology should note that leukemia could mimic common gynecological malignancies such as squamous cell carcinoma of the cervix or endometrial adenocarcinoma. In the case of ovarian mass appearance in women with a history of ALL, the differential includes epithelial carcinoma (middle-aged or older) or germ cell malignancies (younger patients). Thus, a high index of suspicion should be kept because treatment is totally different in the case of ovarian cancer and a totally different treatment is indicated when the ovarian mass is due to leukemic infiltration. As shown in reported cases, misdiagnosis and mismanaging of patients presenting with ovarian mass could directly affect prognosis [8].

Together with the abovementioned data, gynecologists should always keep in mind that the Pap smear is mainly sensitive for SCC and precursor neoplasia. It is far less sensitive for the detection of primary or metastatic Ca, including extramedullary leukemia. Furthermore, radiologic features help to recognize lesions, but only histology and immunohistochemistry can set diagnosis [15].

Finally, cases similar to the one presented will become more common in the future because patients with leukemia exhibit increasingly prolonged survival due to advances in diagnosis and treatment of leukemia.

The Pap test exhibits high sensitivity only for cervical squamous cell carcinomas and cervical intraepithelial lesions which are the relevant carcinoma precursors. In contrast, Pap smears' diagnostic sensitivity is low for any other pathology including the case of leukemia.

Regarding the liquid-based cytology (LBC) preparations, the cons are that malignancy clues are even more subtle compared to conventional smears and the pros refer to the fact that in LBC additional diagnostic test can be performed such as molecular/cytogenetic test and flow cytometry assessments [26].

Therefore, the Pap smear as a screening test is useful in order to alarm physicians when pathologic but nonpathognomonic features are encountered in order to proceed in further diagnostic workup. Pathologic features refer to monotonous inflammatory cells with various degrees of atypia which are nonspecific; however, the patients' history can pave the path towards the exact diagnosis [26].

## Figures and Tables

**Table 1:** Immunophenotyping studies.

Immunohistochemistry	
MIB 1/Ki-67	100% positive
CD34	Positive
CD45	Positive
CD20	Positive
CD79A	Positive
CD10	Positive
